# To what extent naringenin binding and membrane depolarization shape mitoBK channel gating—A machine learning approach

**DOI:** 10.1371/journal.pcbi.1010315

**Published:** 2022-07-20

**Authors:** Monika Richter-Laskowska, Paulina Trybek, Piotr Bednarczyk, Agata Wawrzkiewicz-Jałowiecka

**Affiliations:** 1 Łukasiewicz Research Network–Institute of Medical Technology and Equipment, Zabrze, Poland; 2 Faculty of Science and Technology, University of Silesia in Katowice, Chorzów, Poland; 3 Department of Physics and Biophysics, Institute of Biology, Warsaw University of Life Sciences—SGGW, Warszawa, Poland; 4 Department of Physical Chemistry and Technology of Polymers, Silesian University of Technology, Gliwice, Poland; Bogazici University, TURKEY

## Abstract

The large conductance voltage- and Ca^2+^-activated K^+^ channels from the inner mitochondrial membrane (mitoBK) are modulated by a number of factors. Among them flavanones, including naringenin (Nar), arise as a promising group of mitoBK channel regulators from a pharmacological point of view. It is well known that in the presence of Nar the open state probability (*p*_*op*_) of mitoBK channels significantly increases. Nevertheless, the molecular mechanism of the mitoBK-Nar interactions remains still unrevealed. It is also not known whether the effects of naringenin administration on conformational dynamics can resemble those which are exerted by the other channel-activating stimuli. In aim to answer this question, we examine whether the dwell-time series of mitoBK channels which were obtained at different voltages and Nar concentrations (yet allowing to reach comparable *p*_*op*_s) are discernible by means of artificial intelligence methods, including k-NN and shapelet learning. The obtained results suggest that the structural complexity of the gating dynamics is shaped both by the interaction of channel gate with the voltage sensor (VSD) and the Nar-binding site. For a majority of data one can observe stimulus-specific patterns of channel gating. Shapelet algorithm allows to obtain better prediction accuracy in most cases. Probably, because it takes into account the complexity of local features of a given signal. About 30% of the analyzed time series do not sufficiently differ to unambiguously distinguish them from each other, which can be interpreted in terms of the existence of the common features of mitoBK channel gating regardless of the type of activating stimulus. There exist long-range mutual interactions between VSD and the Nar-coordination site that are responsible for higher levels of Nar-activation (Δ*p*_*op*_) at deeply depolarized membranes. These intra-sensor interactions are anticipated to have an allosteric nature.

## Author summary

The large conductance voltage- and Ca^2+^-activated K^+^ channels from the inner mitochondrial membrane (mitoBK) are modulated by a number of factors, including flavanones like naringenin (Nar). In the presence of Nar the open state probability (*p*_*op*_) of mitoBK channels significantly increases, however the molecular mechanism of the mitoBK-Nar interactions remains still unrevealed. It is also not known whether the effects of naringenin administration on channel gating can accurately mimic the dynamical consequences exerted by other channel-activating stimuli. In order to answer this question, we examine whether the dwell-time series describing the mitoBK channel activity obtained at different voltages (*U*_*m*_) and Nar concentrations are discernible by means of artificial intelligence (AI) methods (k-NN, shapelet learning). The key inferences from the current study are as follows:

- Naringenin most significantly increases the *p*_*op*_ of highly voltage-activated mitoBK channels.
- The Nar binding site and voltage sensor may cooperate allosterically.
- There are some *U*_*m*_- and Nar-specific effects on gating dynamics that allow for a proper AI classification for the majority of input data.
- The local features of the signal seem to be highly stimulus-specific, which can be inferred from a relatively good performance of the shapelet classification method.

## Introduction

### Naringenin as a mitoBK channel modulator

The mitoBK channels can be considered as mitochondrial variants of the large-conductance voltage- and Ca^2+^-activated K^+^ channels (BK) [1]. They play an important physiological role in regulation of metabolism and ATP synthesis (via oxidative phosphorylation) within the inner mitochondrial membrane [1]. Consequently, the mitoBK channels are considered as drug targets. Beside the two generic stimuli that activate the mitoBK channels (membrane depolarization and high availability of Ca^2+^ ions) [2], their open state probability (*p*_*op*_) can be increased in the presence of other factors like mechanical strain [3] or pharmacologically by NS1619 [4], NS11021 [5] or CGS7184 and CGS7181 [6]. In turn, the ionic conduction via mitoBK channels can be effectively inhibited by paxilline (PAX), charybdotoxin (ChTx), iberiotoxin (IbTx), 4-aminopyridine (4-AP) or tetra-ethyl ammonium (TEA), as summarized in [7]. What is, however, worth mentioning, is that many mitoBK channel modulators exhibit a wide spectrum of off-target effects including their cytotoxicity [8]. Thus, further search for the effective and specific mitoBK modulators is needed. It would be also highly valuable to make progress in our understanding of the possible molecular mechanisms of channel—modulator interactions and the interplay between different sensors within the channel that can collectively affect its gating.

Among the potential modulators of mitochondrial channels, flavonoids emerge as cost-effective, non-toxic and easily available candidates for bioactive compounds used in future medicine. Within this group of natural substances, naringenin (Nar) reached a reasonable scientific interest [9, 10] due to its antioxidant, cytoprotective and anti-inflammatory properties. For this sake, naringenin became useful for reducing the risk of oxidative stress and inflammation-mediated processes underlying pathogenesis of many human diseases [11–13].

### In the search of molecular mechanism of the Nar–mitoBK interaction

Considering the molecular mechanism accounting for the observed cellular, and, further, tissue- or even systemic effects of Nar administration, one of the crucial component process is activation of BK and mitoBK channels [5, 14–19]. The molecular mechanism of Nar-mitoBK or Nar-BK interactions remains still not completely understood. Nevertheless, considering the literature reports one can deduce that the binding site for Nar coordination is located within *α* subunits of the channel [14], probably within the gating ring.

A similar level of activation by Nar was observed for the BK/mitoBK channels in different types of cells, where the channel was coordinated with different types of *β* subunits [5, 14–19]. Thus, the presence of auxiliary subunits may exert only a minor indirect effect on channel-Nar interactions.

Moreover, the BK and mitoBK channel variants can be activated by Nar to a similar extent. Accordingly, the presence of the structural differences between the plasma membrane- and mitochondrial channel variants are anticipated not to affect naringenin binding.

It has been also observed that the popular blockers of BK and mitoBK channels can antagonize the effects of Nar, which was described in case of PAX [5, 17, 19], IbTx [5], and TEA [16]. The blockers may be coordinated to the channel residues which determine its transport properties to a higher extent than Nar-binding sites (e.g. coordination of inhibitors impose a physical block of a channel pore).

It is well known that the voltage-sensing domain (VSD) strongly affects the channel gating dynamics through allosteric mechanism [20–23]. The current knowledge about the interactions between the Nar-binding site and the channel gate as well as the possible VSD–Nar-binding site communication is limited. Thus, in this research we will compare the effects of membrane depolarization and the increase of Nar concentration on the mitoBK channel gating.

### A perspective to utilize AI methods in ion channel research

To unravel the details of the molecular mechanism of the interactions between Nar and BK or mitoBK channels, one should carry out a series of calculationally demanding Nar docking by the use of molecular dynamics (MD) methods. Later, its results should be validated in appropriately designed biological models. Despite of the rapid evolution and progress in computational modeling of proteins [24–28] still there exist limitations which preclude them to be used in the studies of conformational dynamics of ion channels on long time scales. Direct tracking of the functional effects exerted by the coordination of modulator like naringenin to a given BK channel variant may stay out of range for modern modeling and simulation techniques. To enable for such studies, some simplifications of the investigated BK-Nar system are needed. They should include indication of anticipated active sites for Nar coordination and description of the possible interferences of naringenin with other channel-regulating stimuli.

In this aim, the single-molecule electrophysiological techniques such as patch-clamp [29] and the following signal analyses can be exploited. In this work, we show that the easily available recordings can be directly utilized to gain some valuable information about the channel’s conformational dynamics. Thus, the aforementioned problems with limitations of simulation techniques can be avoided to a certain degree. We decided to join the advantages of the standard experimental method (patch-clamp) and the techniques of artificial intelligence (AI). The last ones are still progressive in ion channel research. There are only several studies that discuss the use of machine learning approaches to the analysis of the single-channel patch-clamp signals. In the research of Celik et al. the authors present the method of AI utilization to detect and discern the single–molecule events [30] In turn, in the work [31] the use of machine learning methods allows one to classify the patch-clamp signals that correspond to different variants of *α*–*β* BK channel complexes which are typical for particular cell lines. The main advantages of the AI approach in the context of functional analysis of ion channels are that it is directly signal-based and neither requires knowledge about the mechanism of channel gating nor expertise in statistical description of gating kinetics. In a broader context of the research on ion channels, the AI techniques can be also successfully exploited in the channel’s proteomics and genomics, as discussed in [32, 33].

In this work we will answer the question whether voltage- and Nar-activation exert sufficiently specific effects on conformational dynamics that allow them to be discerned from each other by the machine learning (ML) methods. Membrane depolarization changes the location and spatial orientation of VSD within the membrane (so also the exposition of VSD charge). Whereas, naringenin is coordinated within the gating ring (located outside the membrane). In consequence, the structural changes of the channel induced by the voltage activation and the naringenin coordination are evidently disparate. Nevertheless, it is quite interesting whether these stimuli affect the stability of conducting and nonconducting channel’s conformations as well as the overall kinetics of conformational switching in a discernible way.

We use the sequences of dwell-times of subsequent open and closed mitoBK channel states as input data. Such a choice was dictated by the fact that the dwell-time series can be easily constructed from the raw patch-clamp data (which, in turn, have the form of time series of single-channel currents), but they are not biased by the effects of different signal-to-noise ratio at different membrane potentials. Moreover, dwell-time series directly refer to the conformational dynamics of a given channel.

**Fig 1:**
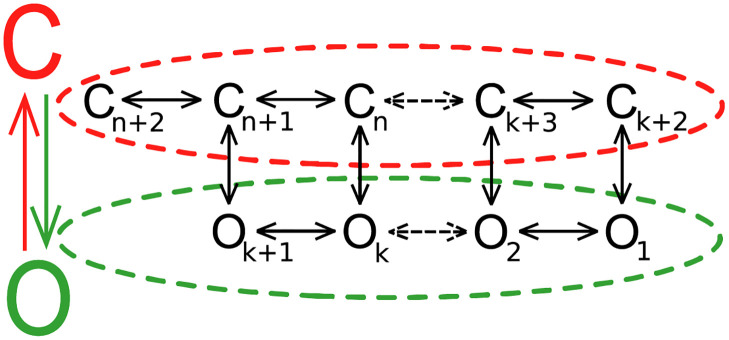
Schematic representation of a general Markovian model of the ion channel gating kinetics [34]. From a simplified point of view the channel can exist in two functionally distinct states: open (O) and closed (C), which are represented by green and red color, respectively. These states can be, in turn, constituted by a discrete number of substates (i.e. (n+2)-(k+2) closed substates *C*_*i*_ and (k+1) open substates *O*_*j*_). The channel’s substates are thought to correspond to its stable conformations. For example, in case of the BK channels, 3-4 open and 5-6 closed substates can represent its kinetics [35]. The possible transitions between channel’s substates are depicted by arrows.

According to the popular Markovian models of the channel kinetics [34], under fixed external conditions there is a limited number of stable channel conformations and possible connections between them (as presented in Fig 1). These are however not known a priori. Depending on the relative stability of the channel conformations and the heights of energetic barriers separating them, they should have different average dwell-times and occur more or less frequently during the recording of channel activity. A single dwell-time brings not sufficiently specific information to recognize to which channel conformation does it correspond. That’s because each channel conformation is described by its own dwell-time distribution [34–37]. Stable conformations must appear multiple times in the patch-clamp recording and there is a limited number of interconnections between them. Therefore, the analysis of dwell-time sequences, which give a temporal description for several acts of switching between channel’s conformations, allows one to describe the gating dynamics in a more unique way. From this perspective, in this work we will perform the classification analysis for sets of dwell-time subseries obtained at different *U*_*m*_s and [Nar]s.

We are convinced that each stimulus should affect the channel’s conformational space in a different manner. Therefore, the relative effects of Nar- and voltage-activation on conformational dynamics will be evaluated by comparing the short excerpts (of 50 points) from the dwell-time series obtained at different different [Nar] and *U*_*m*_ combinations, where the investigated channels will reach a possibly close level of overall activation. Here, mean open state probability *p*_*op*_ is used as the criterion for similarity of channel’s activity level. If the data obtained at different [Nar] and *U*_*m*_ are unambiguously discernible according to the ML methods, these stimuli should affect the conformational dynamics of the channel in a highly specific way. In those terms, a sole dwell-time subseries allows us for identification at what *U*_*m*_ and Nar concentration they were obtained. Accordingly, the number, connectivity and/or stability of the available channel conformations may be independently affected by [Nar] and *U*_*m*_. In opposite case, i.e. when the input signals obtained at different conditions of *U*_*m*_ and [Nar] are indiscernible, it will suggest that there exists a set of predefined stable conformations which are characteristic for channel’s activation level regardless of the type of activating stimulus. In those terms, naringenin binding would allow the channel to mimic the conformational kinetics that usually correspond to its highly voltage-activated state, and vice versa.

Considering the methodology used in the current work to perform the classification of the input signals, we decided to apply the K-nearest neighbors algorithm (k-NN). The k-NN method was commonly used in Time Series Classification (TSC), also in the case of biological signals such as electrocardiography [38] or electroencephalography [39, 40]. According to our previous studies [31] this simple technique can be successfully utilized in case of separation problems dedicated to time series describing ion channel gating.

To get a better insight into the nature of the analyzed problem we also implemented the shapelet-based ML technique. Shapelet is a subsequence of signal, on the basis of which the similarities within the signal can be identified. Based on specific shapelet features the process of time series classification can be evaluated with high accuracy. Since being presented in 2009 [41], the shapelet methodology has gained a wide range of applications also in the field of biosignals [42].

## Results

**Fig 2:**
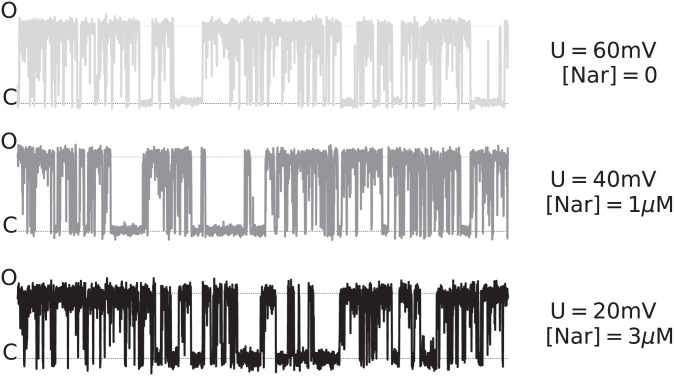
The representative normalized patch-clamp signals (single channel current vs. time) recorded at different membrane potentials *U*_*m*_ and naringenin concentrations [Nar]. The presented traces correspond to the first group of recordings used for ML classification. As one can see, the compared traces have a similar overall characteristics.

The obtained patch-clamp recordings confirmed that naringenin acts as mitoBK channel activator (Table 1). To visualize the basic characteristics of the experimental data, representative normalized signals recorded for different membrane potentials *U*_*m*_ and naringenin concentrations [Nar] are presented in Fig 2. According to the Table 1, the opening-reinforcing effects of naringenin administration are better pronounced in the case of highly voltage-activated mitoBK channels.

**Table 1:** The mean open state probabilities obtained at different membrane potentials and naringenin concentrations. The cells representing the groups of recordings for which the classification analysis by ML was performed are coloured by the same shade of gray. The *U*_*m*_ values are given in [mV].

U_*m*_	control	1 *μ*M Nar	3 *μ*M Nar	10 *μ*M Nar
60	0.59±0.04	0.60±0.03	0.65±0.02	0.71±0.02
40	0.56±0.03	0.58±0.02	0.63±0.02	0.66±0.02
20	0.53±0.05	0.53±0.02	0.58±0.05	0.60±0.02

Considering the merit of this study, the preliminary task of its experimental part was to find such values of membrane potential and naringenin concentration to find groups of recordings which have matching *p*_*op*_ values. Maximal difference of mean *p*_*op*_s was assumed to be 0.01. As presented in Table 1, we have extracted three independent groups for further ML classification and separation; i.e. first group, where *p*_*op*_ ≈ 0.58 constituted by recordings at (U_*m*_=60[mV], [Nar]=0), (U_*m*_=40[mV], [Nar]=1*μ*M) and (U_*m*_=20[mV], [Nar]=3*μ*M), second group, where *p*_*op*_ ≈ 0.60 constituted by recordings at (U_*m*_=60[mV], [Nar]=1*μ*M) and (U_*m*_=20[mV], [Nar]=10*μ*M), and third group, where *p*_*op*_ ≈ 0.66 comprised the recordings obtained at (U_*m*_=60 [mV], [Nar]=3*μ*M) and (U_*m*_=40[mV], [Nar]=10*μ*M).

For all obtained raw experimental data, the corresponding dwell-time sequences were constructed and further analyzed. The results of the signal classification by the use of k-NN and shapelet learning methods are summarized in Table 2.

**Table 2:** The prediction accuracies obtained with the k-NN and shapelet learning method for dwell-time sequences corresponding to different groups of recordings marked by various shades of gray in Table 1.

*U*_*m*_	[*Nar*]	Accuracy k-NN	Accuracy shapelet
60 mV	1 *μ*M	62%	70%
20 mV	10 *μ*M		
60 mV	3 *μ*M	64%	71%
40 mV	10 *μ*M		
60 mV	0 *μ*M	49%	56%
40 mV	1 *μ*M		
20 mV	3 *μ*M		

As one can see, the obtained accuracies indicate that the classification of data is non-random which suggests that some specific features of the dwell-time sequences obtained at different naringenin concentrations and voltages exist. (In case of randomness one should expect the accuracy of 50% for two classes, and 33% for three classes.) In the majority of cases (over 60%), these features allow us to distinguish from each other the dwell-time subseries obtained at different [Nar] and *U*_*m*_, regardless of the fact that the investigated signals exhibit comparable *p*_*op*_s. Nevertheless, these individual features do not manifest themselves often enough to ensure unambiguous discrimination of each dwell-time subseries corresponding to fixed [Nar] and *U*_*m*_. About 30% of analyzed data had not sufficiently different characteristics to distinguish them from each other unequivocally. One can interpret that in terms of the existence of some common predefined paths of conformational switching regardless of the type of activating stimulus.

To further investigate the problem, we have also performed the ML classification for the experimental data obtained at two different naringenin concentrations and fixed membrane potential, and vice versa. The results are presented in Table 3. In the first case, the leading factor responsible for the increase of open state probability (Δ*p*_*op*_ = 0.12) is administration of naringenin. Here, the compared groups of recordings were obtained at fixed membrane potential *U*_*m*_ = 60 mV in absence of naringenin and when this modulator was introduced [Nar] = 10 *μ*M. In the second case, one can observe a similar level of open state promotion (Δ*p*_*op*_ = 0.11) which was, in turn, related to the raising value of membrane potential (from 20 mV to 60 mV) at fixed [Nar] = 10 *μ*M. According to the results in Table 3, it is not clear what is the main factor (*U*_*m*_ or Nar) affecting efficiency of data classification. There are, however, differences between the performance of separation algorithms for the data referring to the Nar- and voltage-activation. The k-NN results allow for reaching relatively high prediction accuracy for channel activation by Nar coordination. It may suggest that naringenin-activation mostly shapes the large-scale features of the channel gating. In turn, voltage-activation is supposed to exert significant effects on local features of the mitoBK channel gating, according to the better accuracy of the shapelet analysis.

**Table 3:** The prediction accuracies obtained with the k–NN and shapelet methods for the groups of experimental data representing different types of channel activating stimuli (i.e. [Nar] and *U*_*m*_).

*U*_*m*_	[*Nar*]	Accuracy k-NN	Accuracy shapelet
60 mV	10 *μ*M	76%	62%
60 mV	0 *μ*M		
60 mV	10 *μ*M	66%	82%
20 mV	10 *μ*M		

**Fig 3:**
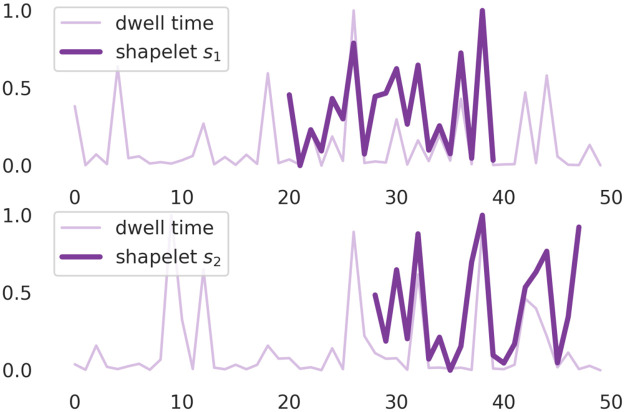
Two exemplary samples of dwell time series of length *N* = 50 obtained from the analysis of patch–clamp recordings corresponding to [Nar] = 0 *μM* (upper figure) and [Nar] = 10 *μM* (lower figure) and the same mebrane potential *U*_*m*_ = 60 mV. Both sequences are presented alongside with the shapelets *s*_1_, *s*_2_ found by the machine learning algorithm of length 20 which best match the shape of signal.

**Fig 4:**
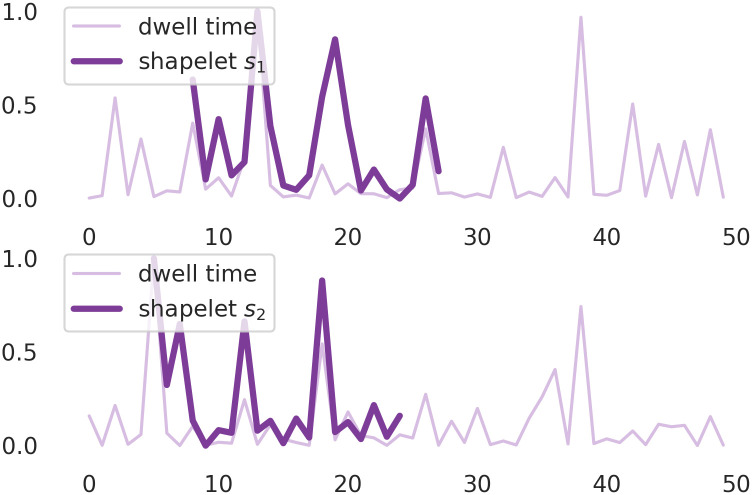
Two exemplary samples of dwell time series of length *N* = 50 obtained from the analysis of patch–clamp recordings corresponding to *U*_*m*_ = 20 mV (upper figure) and *U*_*m*_ = 60 mV (lower figure) and the same naringenin concentration [Nar] = 10 *μM*. Both sequences are presented alongside with the shapelets *s*_1_, *s*_2_ found by the machine learning algorithm of length 20 which best match the shape of signal.

To further inspect the problem of differences in accuracy of the shapelet classification of signals recorded at different naringenin concentrations and membrane potentials, in Figs 3 and 4 we have presented those parts of the signal which were recognized as its most characteristic parts (shapelets). The shapelets are drawn on the parts of the signal, for which the minimal distance in the transformation space was found (Figs 3 and 4). As one can see in Fig 3, the shapelets that discriminate best the extreme regimes of Nar concentrations refer to the dwell-time sequences of relatively large internal variability. Whereas the shapelets corresponding to the separation of data corresponding to different voltage-activation levels seem to describe the entrance/exit path to/from long-lasting states via the relatively short-lasting ones (Fig 4).

**Fig 5:**
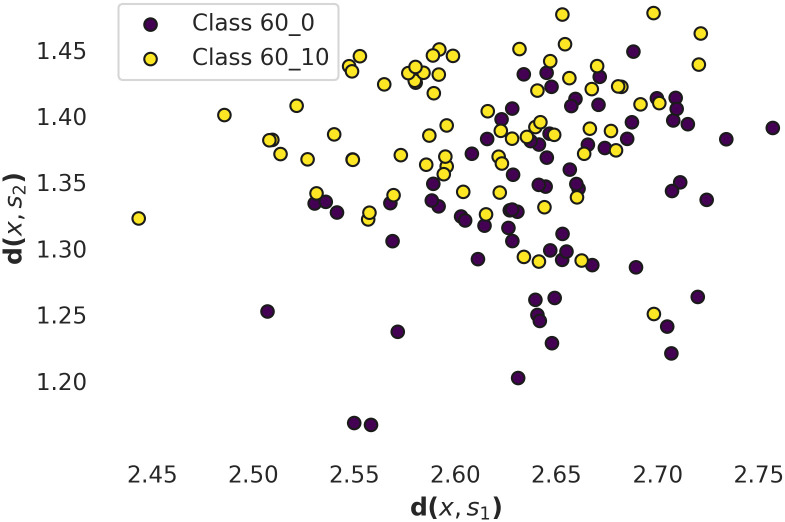
The shapelet–transform representation of the input data describing mitoBK activity obtained at naringenin concentration of [Nar] = 0 *μM* (purple dots) and [Nar] = 10 *μ* M (yellow dots) and the same membrane potential *U*_*m*_ = 60 mV. Graph represents the distances *d*(*x*, *s*_1_) and *d*(*x*, *s*_2_) of all dwell time samples included in the dataset to two representative shapelets presented in Fig 3 calculated according to Eq 3.

**Fig 6:**
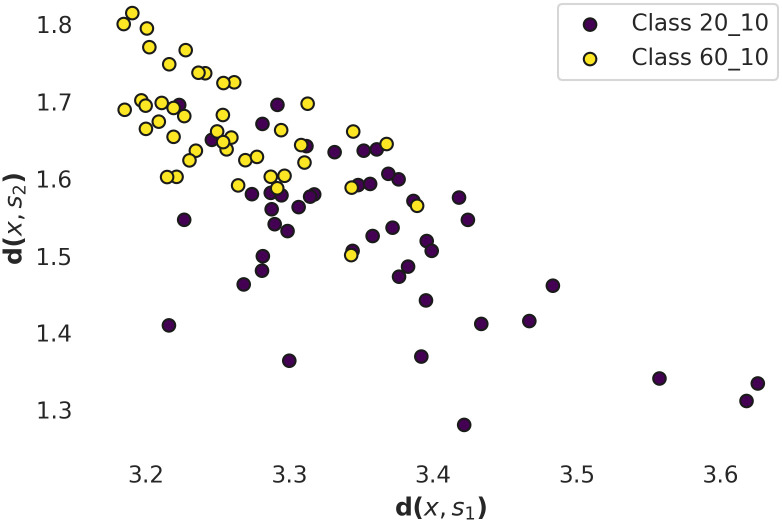
The shapelet–transform representation of the input data describing mitoBK activity obtained at membrane voltages *U*_*m*_ = 20 mV (purple dots) and *U*_*m*_ = 60 mV (yellow dots). Graph represents the distances *d*(*x*, *s*_1_) and *d*(*x*, *s*_2_) of all dwell time samples included in the dataset to two representative shapelets presented in Fig 4 calculated according to Eq 3.

In Figs 5 and 6 there are presented relative distances between the points representing experimental data obtained at different [Nar] and *U*_*m*_ in shapelet–transform space. In Fig 5 one can observe relatively high overlapping of points corresponding to [Nar] = 0 and [Nar] = 10 *μ*M. In that case 38% of points overlap and consequently may be misclassified. The points corresponding to the different *U*_*m*_s (*U*_*m*_ = 20mV and *U*_*m*_ = 60mV) are relatively well separated (only 18% of points overlap). Thus, one can infer that at a relatively short observation scale (of the *N*_*s*_ order of magnitude) the *U*_*m*_ predefines the channel gating dynamics and leads to occurrence of the voltage-characteristic sequences of dwell-times. Whereas the Nar coordination has only an accessory effect on the short-scale signal’s characteristics.

## Discussion

The current study allows us to conclude that naringenin activates the mitoBK channels in a voltage-dependent manner (Table 1) and its coordination may frequently exert an observable specific effect on channel gating. The results presented in Table 1 and in the work of Kicińska et al. [5] indicate shifting of voltage activation curve (*p*_*op*_(*U*_*m*_)) toward more negative potentials in presence of naringenin. Moreover, Nar also promotes channel opening in a voltage-dependent manner. Greater increments of *p*_*op*_s for raising [Nar] are observed at highly depolarized membranes (Table 1). It may suggest that voltage sensor activation and naringenin binding can influence each other. An interesting question to understanding mitoBK channel function is how the protein domains involved in sensing stimuli (*U*_*m*_, Nar etc.) and the channel pore opening communicate.

To address the molecular picture of this phenomenon, we have briefly inspected whether there exists a possibility that the Nar-binding affects the position and/or orientation of the voltage sensing domain of mitoBK channel. In that aim, we had to use “auxiliary” patch-clamp recordings obtained at negative voltages (-40 ÷ -60 mV) for which the *p*_*op*_s were near to zero (*p*_*op*_ ≤0.04), which were obtained at different naringenin concentrations. Then, we estimated the apparent gating charge according to the formula [43]:

$$\begin{array}{c} q_{s}\left(U_{m}\right)=kT\frac{dln(p_{op}\left(U_{m}\right))}{dU_{m}} \end{array}$$ (1)

where k is Boltzmann constant, T denotes temperature. For all analyzed data the obtained values of apparent gating charge were comparable and reached *q*_*s*_(-50 mV) ≈ 1.9*q*_0_ regardless of naringenin concentration. This result was in agreement with typical values for BK, so also for mitoBK, channels [44]). It suggests no effect of Nar-coordination on the observable *q*_*s*_.

Furthermore, to inspect the possible intra-sensors’ interactions we would like to refer to the popular Markovian models of the channel gating [34–37] (Fig 1). According to these approaches, the experimental dwell-time distributions may be used to estimate the minimal number of substates that represent the kinetics of the investigated channels. Each substate of the channel constitutes an exponential component to the distribution of open or closed interval durations. In the case of our analysis, 2 open and 3 closed substates modeled the channel gating regardless of the naringenin concentration and membrane potential. It is in agreement with the reduced model systems describing the BK (and mitoBK) channel kinetics at fixed external conditions found in literature [35]. The number of component exponentials is only a rough estimation of the number of substates in the Markovian model that may represent the stable channel conformations. Nevertheless, this kind of analysis allows us to gain a certain depiction of the channel gating characteristics. It suggests that the mitoBK channel may exist in a discrete number of conformational states that are in thermodynamic equilibrium in the absence of naringenin. The presence of that modulator ought to merely shift the equilibrium between the available stable conformations, selectively fostering the ones for which it displays the highest affinity. What is important, it seems that the number of available channel conformations does not change with Nar coordination.

The type of naringenin impact on channel’s behaviour is common for other ligands that regulate BK/mitoBK channel gating and activation through an allosteric mechanism, as e.g. divalent cations (Ca^2+^, Mg^2+^) or heme [20–23, 44–46]. The voltage sensor activation promotes channel opening also allosterically [21]. What can be hypothesized about the voltage-sensor–naringenin binding site communication? There is a diametrically opposed molecular mechanism of the voltage activation and the naringenin coordination to the mitoBK’s channel gating ring. Nevertheless, there exists an observable cooperation-effect between voltage sensor activation and naringenin binding that may enhance the tendency of the channel gate to open. Thus, it is suspected that the communication between the VSD and Nar-binding site has also an allosteric nature.

An analogous type of interaction is anticipated in case of Ca^2+^ and Nar-binding sites, according to the results obtained in [5]. In that work the effects of Nar coordination on channel gating were Ca^2+^-dependent. Namely, naringenin activeted the channel to a highest degree in a low [Ca^2+^] regime. Thus, our investigations together with the other comprehensive structural and functional studies emphasize that the BK and mitoBK channels carry a variety of allosteric modulatory sites in addition to the main categories of regulatory and biologically active sites. The mentioned regulatory sites mediate nested hierarchies of allosteric regulations which earn interest as the potential targets for drug design [47].

Going back to the main focus of this work, here we compared the effects of voltage- and Nar activation on channel gating by means of ML. For over 60% of dwell-time sequences, different combinations of naringenin concentrations and membrane potentials used in experiments affect the channel gating in such an unique way that allow for proper recognition of the channel’s conformational diffusion at given [Nar] and *U*_*m*_ (see Table 2). Still, however, for a significant part of input data are indiscernible for ML classifications algorithms. These results suggest the existence of a repetitive part of the channel gating dynamics for which the single-channel signals have similar characteristics regardless of the type of channel-activating stimuli that is responsible for reaching a certain level of the open state probability.

Comparing the execution effects of distance-based classification method (k-NN) and frequency-based classification method (shapelet), the results shown in Tables 2 and 3 suggest that the shapelet learning method exhibits better performance than k-NN in most cases. The possible explanation should refer to the fact that the shapelet analysis directly detects the similarity between the representative subsequences (subshapes) within the signal. Colloquially speaking, it goes into details of the signal’s structure complexity. During data classification, we analyzed the dwell-time subseries of *N* = 50 elements. In turn, the shapelet length was set at *N*_*s*_ = 10. Thus, the shapelets refer to a relatively short sequence of events which are mostly distinctive for a given group of data. In turn, the well-known drawback of the k-NN is that this method may perform poorly with noisy series as the ones we analyze in this work. Moreover, the k-NN method refers to global signal characteristics, because it is calculated as Euclidean distance between the compared subseries. The analyzed data can be considered as a record of switching between the channel’s stable conformations that correspond to *N* = 50 subsequent open and closed states. As mentioned before, in gating dynamics the number of channel’s conformations and the number of possible interconnections between them are limited. In accordance with our simple analysis of channel’s kinetics, voltage- and Nar-activation didn’t significantly influence the number of available channel’s conformations in the investigated regimes of *U*_*m*_ and [Nar]. Nevertheless, for each analyzed subseries the starting conformation, the energetic landscape for conformational space, and, finally, the exact path of conformational changes are unknown. The analysis is even more complicated due to the fact that each conformation is described by its own exponential dwell-time distribution. For this sake, the k-NN results are highly biased with the aforementioned issues.

Referring to the results of the shapelet analysis presented in Table 3, Figs 5 and 6, they suggest that on a relatively short observation scale the *U*_*m*_ is an activating factor that shapes the internal structure of the signal in a more specific way. Increasing the value of *U*_*m*_ results in occurrence of new voltage-characteristic sequences of channel’s dwell-times. Their occurrence gives evidence on repetitivity of some characteristic series of popular channel conformations of given average lifespans which are connected with each other at fixed *U*_*m*_. The effects of naringenin coordination seem to exert a minor effect on the gating dynamics on a short time scale, due to the lower prediction accuracy of the shapelet classification for the dwell-time series at [Nar] = 10 *μ* M and in absence of this modulator (Table 3).

## Conclusions

Artificial intelligence methods are still gaining popularity in the investigation of ion channels activity. In this work we manifested the utility of the AI techniques in the analysis of patch-clamp signals and evaluation of the relative effects of different stimuli on the channel gating. We took the advantages of artificial intelligence (k-NN, shapelet learning) analysis to compare the effects of membrane depolarization and the increase of Nar concentration on the temporal characteristics of channel’s conformational dynamics. The obtained results suggest that both stimuli affect the structural complexity of the analyzed signal. There exist stimulus-specific features of the signal that allow distinguishing over 60% of analyzed dwell-time sequences obtained at different *U*_*m*_s and [*Nar*]s. Our brief kinetic inspection allows us to hypothesize that membrane depolarization and Nar-coordination does not lead to changes in the number of available channel conformations, but rather affects the energetic landscape of channel’s conformational space. Thus, the statistics of the dwell-times of channel states can differ with *U*_*m*_ and [Nar], as well as the structure of the repetitive temporal patterns of switching between channel’s conformations. On a short observation scale typical for the shapelet method, the dwell-time series of channel states are predominantly shaped by the voltage sensor interactions with the channel gate and there exist some *U*_*m*_-characteristic sequences of dwell-times. In this regard, only an additional accessory effect is exerted by the Nar-binding site. Between the VSD and the Nar-coordination site there exist long-range mutual interactions that are responsible for higher levels of Nar-activation at deeply depolarized membranes, which are anticipated to have an allosteric nature.

## Materials and methods

### Cell culture

In this study we used the commercially available stable human endothelial cell line EA.hy926, that was originally derived from a human umbilical vein. The cell culture was carried out in Dulbecco’s modified Eagle’s medium (1000 mg/L D-glucose) supplemented with 10% fetal bovine serum (FBS), 1% L-glutamine, 2% hypoxanthine- aminopterin- thymidine (HAT), 1% penicillin/streptomycin in a humidified 5% *CO*_2_ atmosphere, at 37 °C. The cells were reseeded every third day. The presence of mitoBK channels in the inner mitochondrial membrane was previously described in [48].

### Mitochondria and mitoplast preparation

Mitochondria and subsequent mitoplast were prepared by differential centrifugation and hypotonic swelling as previously described in [48, 49]. In brief, after isolation of mitochondria from the endothelial cells, they were incubated in a hypotonic solution containing 5 mM HEPES, 100 *μ*M CaCl_2_, pH 7.2 for approximately 1 min. After that a hypertonic solution (750 mM KCl, 30 mM HEPES, and 100 *μ*M CaCl_2_, pH 7.2) was subsequently added up to full restoration of the isotonicity of the medium (n = 90). A fresh mitoplast was used for each/repeating patch-clamp experiment.

### Electrophysiology

Patch-clamp experiments were performed in single-channel mitoplast-attached mode at room temperature, as described in [48, 49]. The pipette of borosilicate glass had a resistance of 10–20 ΩM (Harvard Apparatus GC150-10, Holliston, Massachusetts, USA). The patch-clamp pipette solution was isotonic and contained 150 mM KCl, 10 mM HEPES, and 100 *μ*M CaCl_2_ at pH 7.2. Naringenin was added as dilution in the isotonic solution via perfusion system as previously described in [5].

The current was recorded using a patch-clamp amplifier Axopatch 200B. The currents were low-pass filtered at a corner frequency of 1 kHz. We used experimental time series of 20 seconds recorded at sampling frequency 10 kHz. At each value of membrane potential and naringenin concentration, we recorded time series of single mitoBK channel currents using 3–7 independent mitoplast patches.

### Kinetic analysis

For each patch-clamp recording the threshold current value *I*_*TR*_ is found using the algorithm described in [31]. The *I*_*TR*_ separates the currents corresponding to open (conducting) and closed (non-conducting) channel states. Based on the relation between each recorded single-channel current value and *I*_*TR*_, the open state probability (*p*_*op*_) is determined. After that the dwell-time series of successive open and closed states is constructed as well as the corresponding dwell-time distributions for each recording. The dwell-time series is a series of durations of the subsequent open/closed channel states.

According to the popular picture of BK activity (so also appropriate for their mitoBK counterparts) as a Markovian process [34–37], basing on the experimental dwell-time distributions one may estimate the minimal number of states in Markovian model (Fig 1) that represents the kinetics of the investigated channels. Assuming that each substate within open/ closed manifold of channel states (e.g. states *O*_1_ − *O*_*n*_ in Fig 1) constitutes an exponential component to the distribution of open/closed state sojourns (*f*(*t*)), it takes the form:

$$\begin{array}{c} f\left(t\right)=\sum_{i=1}^{N}\frac{a_{i}}{\tau_{i}}exp\left(-\frac{t}{\tau_{i}}\right) \end{array}$$ (2)

where N is a number of substates within a manifold of a given macrostate (open or closed), a_*i*_ describes the fraction of the total area of the dwell-time distribution contributed by the i-th exponential, *τ*_*i*_ is the time constant of the i-th exponential.

The estimated number of substates (*N*) is in fact the minimal value, due to the fact that some substates may not be detected because they have so close values of time constants to the other ones that they overlap within the distribution. Nevertheless, calculation of the number of summed exponentials and the corresponding parameters (areas *a*_*i*_ and time scales *τ*_*i*_) allows us to gain a certain depiction of the gating kinetics of the channel under investigation.

### ML

Our investigation of different patch–clamp recordings involves the usage of machine learning (ML) algorithms. Such methods turned out to be effective in the ion channel analysis. They are able to identify ion channels and their types [33, 33], predict the ion channel conductance based on cardiac action potential shapes [50], detect the single–molecule events [30] or classify the ion channel currents corresponding to different cell lines [31].

In this study, we aim to verify the performance of ML methods used for the classification of mitoBK channels obtained at various membrane potentials *U*_*m*_ and naringenin concentrations [*Nar*].

Our first attempt of the data analysis consists of application of the standard k–NN (k–nearest neighbors) technique with an euclidean distance metric (where *k* = 5). The choice of this algorithm is motivated by its well–documented time–series classification efficiency [51–54]. Moreover, this method achieves excellent results in recognizing the mitoBK channels corresponding to different cell lines basing on results obtained from the patch–clamp experiment [31]. The k–NN classifier is also fast, easy to implement and does not need extensive parameter tuning [55].

The general scheme illustrating classification process within the k–NN algorithm for one data point consists of the following steps:

1. Selection of the number of neighbors *k* (which should be properly tuned as it gives balance between over– and under– fitting)
2. Calculation of the euclidean distance between the new data point intended for classification and the training samples
3. Choice of the *k*–nearest training points and assignment to the appropriate category by the majority voting.

Although k–NN is fast and its application yields in many cases excellent accuracies it does not give much insight into data. In the context of our study, it does not allow us to investigate whether the analyzed ion channels’ activators exert a specific effects on the structure of dwell-time sequences. In order to address this problem, we decided to apply the series shapelet method [56] which has already been proved to be effective in the domain of medical and health informatics [57–59].

The shapelets S are defined as subsequences of the time series $T_{i}$ that are maximally representative of a class. In general, to find such characteristic patterns in the time–series we would have to consider all subseries of length *l* (*l* can be chosen arbitrarily) as potential candidates for a shapelet $S_{k}$. For a given candidate one calculates its distance to the whole series $T_{i}$, which is defined as [56]:

$$\begin{array}{c} M_{i,k}=\min_{j=1,\ldots J}\frac{1}{l}\sum_{i=1}^{l}{\left(T_{i,j+l-1}-S_{k,l}\right)}^{2}. \end{array}$$ (3)

The metric *M*_*i*,*k*_ defined in Eq 3 is simply interpreted as the euclidean distance of a shapelet $S_{k}$ to its most similar segment in $T_{i}$.

To significantly accelerate our computations, instead of searching for the optimal shapelets among all candidates, we use the method proposed in [60]. This technique can be summarized in two steps:

1. Start with the random shapelet
2. Learning the optimal shapelet by minimizing the classification loss function.

This method works as follow. Let us assume, for the sake of simplicity, only two classes of the time series. Then, the corresponding labels can be either 0 for time subsequences belonging to the first class or 1 for the samples attributed to the second class. The label for time sample *i* is denoted as **y**_*i*_.

The prediction $\hat{y_{i}}$ for sample *i* is calculated with the logistic regression model as:

$$\begin{array}{c} \hat{y_{i}}=b_{i}+\sum_{j=1}^{J}M_{i,k}W_{k}, \end{array}$$ (4)

where *b*_*i*_, *W*_*k*_ (called *bias* and *weight*) are free parameters of the model learnt in the process of training and coefficients *M*_*i*,*k*_ are defined in Eq 4.

The parameters *b*_*i*_, *W*_*k*_, and indirectly shapelets *S*_*k*_ are found through the minimization of the loss function:

$$\begin{array}{c} L\left(y,\hat{y}\right)=-y\;\text{ln}\;\sigma \left(\hat{y}\right)-\left(1-y\right)\;\text{ln}\;(1-\sigma \left(\hat{y}\right)) \end{array}$$ (5)

using the stochastic gradient descent algorithm (SGD). Further details of the implemented algorithm can be found in [60].

Within the below–described approach, the number of shapelets *n* and their lengths *l* are chosen arbitraily. We found that the optimal choice for our dataset is *n* = 10 and *l* = 20. Furthermore, in order to minimize the loss function given in Eq 5 we decided to use Adam optimizer with the learning rate *lr* = 0.01 along with the L2 weight regularizer of value 0.001.

**Fig 7:**
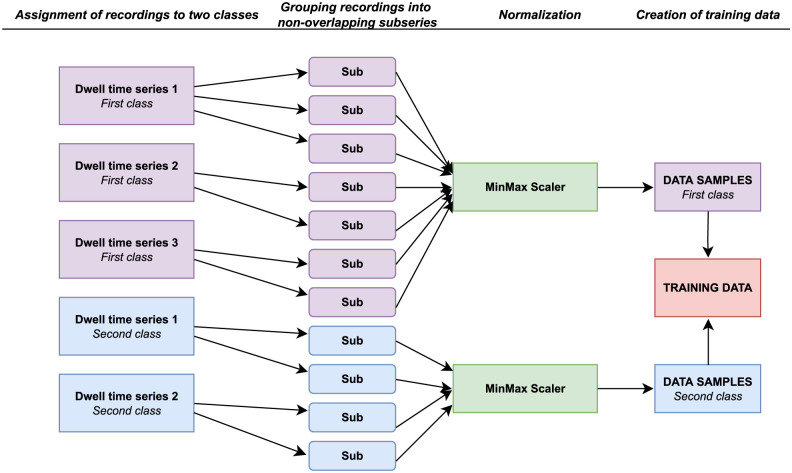
The preprocessing stage of the raw dwell-time series. Before feeding the data into a machine learning algorithm the dwell-time series are assigned to the appropriate classes. They are then divided to the non–overlapping subseries of 50 elements, grouped together and globally normalized to the range [0, 1]. At the end, data samples coming from all classes are combined to create the ultimate dataset.

Before feeding the data into the ML algorithm (either k-NN or *learning–shapelet method*) we preprocess it according to the procedure summarized in Fig 7. At the beginning each dwell-time series (typically of length 700) is divided into the smaller, non–overlapping subseries consisting of 50 points each. Afterwards, all subseries belonging to the same class are normalized into the range [0, 1]. In the final step, such prepared samples are combined to create the ultimate dataset.

In both cases, the performance of the ML algorithm is verified using the *k–fold cross-validation* technique, which allows us to avoid the over-fitting problem. Within this method, the analyzed dwell-time series are divided into the testing and training datasets in the random manner. We split the data around 20%*vs*.80% between testing and training sets. This procedure is repeated 10 times. In each iteration, the accuracy (*Acc*) of an algorithm is evaluated as the ratio of the number of correctly predicted dwell-time series to all samples in a dataset. In the case of binary classification we use the below–presented formula:

$$\begin{array}{c} Acc=\frac{TP+TN}{TP+FP+TN+FN}\cdot 100\%, \end{array}$$ (6)

where *TP* and *TN* denote the number of correctly identified samples in the first class (*TP*) and in the second class (*TN*), whereas *FP* and *FN* stand for the misclassified subseries belonging to the first and second class, respectively. Note that, the extension of this formula to the three groups classification problem is trivial.

The overall performance score is an average of the accuracy scores as calculated across 10 test folds.

Additionally, apart from classification, we use the *learning–shapelet* technique in order to reduce the dimensionality of our samples and visualize them in the 2–dimensional space. For this purpose, we calculate the distances **d**_**1**_ and **d**_**2**_ of each dwell-time subseries (according to Eq 3) to two chosen shapelets **s**_**1**_ and **s**_**2**_ and treat these distances as the new coordinates in the distance–transformed space. Note that, this procedure is applied only for the visualization purposes and does not have any impact on the results presented in Tables 3 and 2.

All calculations concerning *learning shapelet* and k–NN methods were conducted with the use of the tslearn and scikit-learn Python libraries [61, 62].
